# Maternal Betahistine Exposure and Pregnancy Outcomes: A Retrospective Cohort Study

**DOI:** 10.7759/cureus.96451

**Published:** 2025-11-09

**Authors:** Doua Alsaad, Afif Ahmed, Wessam Elkassem, Ahmed Moursi, Fathima Minisha

**Affiliations:** 1 Pharmacy, Hamad Bin Khalifa Medical City, Hamad Medical Corporation, Doha, QAT; 2 Pharmacy, Hazm Mebaireek General Hospital, Hamad Medical Corporation, Doha, QAT; 3 Obstetrics and Gynecology, Women's Wellness and Research Center, Hamad Medical Corporation, Doha, QAT

**Keywords:** betahistine, fetus, pregnancy, safety, teratology

## Abstract

Background

In the general population, betahistine has a good safety profile. However, there is insufficient data on betahistine use during pregnancy; safety and teratogenic risks are yet to be evaluated. The study aimed to review pregnancy outcomes in women who were exposed to betahistine during their pregnancies and identify the rate and pattern of major malformation.

Material and methods

A descriptive retrospective cohort study was conducted. Pregnant women who were prescribed betahistine from Hamad Medical Corporation hospitals between 1st-April-2020 and 31st-March-2022 and had their delivery or abortion at the Women’s Wellness and Research Center, Qatar, were included. Patients were followed up until the occurrence of the study outcomes (delivery or abortion), with an additional two months of follow-up for the baby if data were available. Ethical approval was obtained from the Medical Research Center (MRC-01-23-223).

Results

Forty pregnant women were included, with 42 baby outcomes (including two sets of twins). Betahistine daily dose ranged between 16 mg and 48 mg, with 75% of patients receiving the higher dose of 48 mg. The majority of patients received betahistine during the first trimester (90%) and for a short duration of ≤ 14 days (80%). Six pregnancies ended with spontaneous miscarriage (14.3%), and 34 pregnancies resulted in 36 live babies (85.7%). Four babies (11.1%) were preterm (delivered before 37 weeks of gestation), and four babies had a weight < 2500 g at birth (11.1%). Thirty-one babies had normal outcomes (86.1%), five babies had minor malformations (13.9%), and none had major malformations. These minor malformations were mild pyloric stenosis, bilateral positional talipes, displaced ear, umbilical hernia, hydrocele, tongue tie, and bilateral talipes calcaneus.

Conclusions

The current study does not suggest an increased rate of major congenital malformations following in-utero betahistine exposure. However, the small sample size and retrospective nature of the data warrant caution in result interpretation and do not confirm its safety for use in pregnant women. Avoidance of unnecessary medication during pregnancy is advisable whenever possible.

## Introduction

Betahistine is commonly used for the management of balance disorders and vertigo symptoms. It is a histamine analog with a partial agonist of the histamine H1 receptor and a strong antagonist of the histamine H3 receptor. The exact mechanism of action is not yet understood; however, its multifactorial mode of effect is supposed to restore proper balance and reduce symptoms of vertigo [1-5].

Vestibular disorders are commonly seen in pregnant women, such as vestibular migraine, benign paroxysmal positional vertigo, and Meniere’s disease [6,7]. Vascular and hormonal changes may contribute to the pathogenesis of vestibular dysfunction from the time of conception until the end of pregnancy [8,9]. A cross-sectional study revealed that 37.1% of pregnant women reported a history of vertigo [10]. The high incidence of vertigo among pregnant women necessitates highlighting this symptom in clinical settings and providing proper management.

In the general population, betahistine has a good safety profile [11,12]. However, there is insufficient data on betahistine use during pregnancy. Safety and teratogenic risks are yet to be evaluated in this patient population. The datasheet for betahistine dihydrochloride states that animal studies show no evidence of direct or indirect harmful effects on reproductive toxicity at therapeutic doses relevant to clinical use [13]. A case series study conducted by Buharalioglu et al. investigated the pregnancy outcomes following in utero exposure to betahistine. The study included 20 live births, with 17 babies showing normal outcomes, two babies having minor malformations, and one baby having a major malformation. The results of this study do not suggest an increased risk of major malformation with betahistine use during pregnancy; however, the authors advise interpreting these results with caution due to the small sample size and the data collection method. Furthermore, the authors concluded that it would be wise to avoid betahistine use during pregnancy [8].

The lack of comprehensive data about the safety of betahistine for both pregnant women and the exposed fetuses necessitates conducting further studies to fill this gap in knowledge. Furthermore, understanding the potential risks associated with betahistine use during pregnancy helps healthcare providers in making informed decisions about its use. The aim of the current study was to review the pregnancy outcomes of women who were exposed to betahistine during their pregnancies and identify any congenital malformations or adverse physical effects in the exposed fetus.

## Materials and methods

Study design

A descriptive retrospective cohort study was conducted to evaluate outcomes for pregnant women who received betahistine. The study reviewed pregnant women who were prescribed betahistine over a two-year period, from April 1st, 2020, to March 31st, 2022. These patients were followed up until the end of pregnancy, either by delivery or abortion, with two months of follow-up for the baby if data were available.

Study setting and patient population

Pregnant women who were prescribed betahistine from Hamad Medical Corporation (HMC) hospitals during the specified study period and had their delivery or abortion at Women’s Wellness and Research Center (WWRC), Qatar, were included in the study. Pregnant women with unclear documentation, unknown outcomes, or exposure to known teratogens were excluded.

Study outcomes

The rate and pattern of major malformation were the primary outcomes for this study. The secondary outcomes were the rates of miscarriage, stillbirth, preterm birth, and low birth weight. The Malformation Coding Guides of European Surveillance of Congenital Anomalies (EUROCAT) were used to classify major and minor malformations [14]. "Preterm baby" refers to a birth before 37 weeks of gestation, and "low birth weight" indicates a weight at birth of < 2500 grams. Miscarriage describes the spontaneous loss of a pregnancy before 20 weeks of gestation, while intrauterine fetal death (IUFD) and stillbirth refer to pregnancy loss after 20 weeks of gestation. The rate of malformation was calculated for live births only, because data on major congenital malformations for miscarriages were lacking.

Data collection

A standardized data collection sheet was developed utilizing a literature review [8,13]. Variables about demographics, maternal characteristics, and maternal and neonatal outcomes were obtained. Data collection was aimed at identifying any congenital malformations or adverse physical effects discovered during pregnancy or at birth. Where data was available, baby files were reviewed up to two months of age. Variables were collected for demographics (age, gravida, para, chronic diseases, current pregnancy diseases, previous miscarriage or fetal death, previous baby with malformation, known documented exposure), betahistine use (dose, duration, time of exposure, indication), and pregnancy outcomes (gestational age at delivery, mode of delivery, baby gender, baby weight, baby health at delivery and after two months). To standardize the pattern of data collection, a key description for variables was inserted in the data collection sheet as needed. Data collection was done by the study researchers. Disagreements were resolved by discussion and/or by consulting further research if needed.

Ethical considerations

Patient identification information was coded. A waiver for informed consent was granted for the retrospective data collection. The study has been approved by the Medical Research Center (MRC-01-23-223), HMC.

Statistical analysis

Simple descriptive analysis using percentages and ranges was used to describe patients’ characteristics, betahistine use, rate of malformation, and other pregnancy outcomes. The rate of malformation was calculated based on the number of live babies as a denominator to account for twin pregnancies. The statistical analysis was carried out using an Excel sheet (Redmond, USA).

## Results

A total of 40 pregnant women were included in the analysis, resulting in 42 baby outcomes (including two sets of twins). Patients’ characteristics are summarized in Table 1. The majority of patients were younger than 35 years (n=34, 85%). Around 17.5% of the patients had gestational diabetes in the current pregnancy, corresponding to seven patients. In 19 patients (47.5%), there was a history of one or more miscarriages. There was no record of malformation history in previous pregnancies in the patients' files.

**Table 1:** Maternal characteristics and obstetric history* * Data for 40 pregnant women, IUFD: intrauterine fetal death

Variables	Number (%)
Age	
< 35	34 (85)
≥ 35	6 (15)
Medical conditions	
Gestational diabetes	7 (17.5)
Diabetes mellitus type 2	1 (2.5)
Chronic hypertension	1 (2.5)
Asthma	6 (15)
Hypothyroidism	3 (7.5)
Gravida	
1	6 (15)
2	5 (12.5)
>2	29 (72.5)
Para	
0	8 (20)
1	10 (25)
>1	22 (55)
Current pregnancy	
Spontaneous	40 (100)
Singleton	38 (95)
Twin	2 (5)
Previous miscarriage	
1 or more	19 (47.5)
Previous IUFD/stillbirth	
1 or more	1 (2.5)
Previous baby with malformation	
Not documented	40 (100)

Use of betahistine is described in Table 2. The daily dose of betahistine ranged from 16 mg to 48 mg, with 30 patients (75%) receiving the higher dose of 48 mg. Most of the patients were prescribed betahistine during the first trimester (n = 36, 90%) and for a short duration of 14 days or less (n = 32, 80%). All patients received betahistine for benign paroxysmal positional vertigo (BPPV) and dizziness.

**Table 2:** Use of betahistine* * Data for 40 pregnant women, ** One patient received two prescriptions (at two weeks and five weeks gestation), BPPV: Benign paroxysmal positional vertigo

Variables	Number (%)
Route of administration	
Oral	40 (100)
Daily dose of betahistine	
16 mg	1 (2.5)
24 mg	7 (17.5)
32 mg	2 (5)
48 mg	30 (75)
Gestational week at time of exposure (prescribing time)	
1st trimester	36 (90)
Prior to 4- weeks’ gestation**	27
Between 4- and 12-weeks’ gestation**	10
2nd and 3rd trimester	4 (10)
Duration of exposure	
1 – 14 days	32 (80)
14 – 32 days	8 (20)
Indication for betahistine use	
BPPV/Dizziness/Vertigo	40 (100)

Details for pregnancy and baby outcomes are presented in Table 3. Out of the 42 pregnancy outcomes, six pregnancies ended with spontaneous miscarriage (14.3%), and 34 pregnancies resulted in 36 live babies (85.7%), all of whom were included in the analysis. Follow-up data were available for 31 babies who had at least one visit to the hospital or emergency department within the first two months of life (86.1% of live babies). No intrauterine fetal death or stillbirth cases. Four babies (11.1%) were preterm (delivered before 37 weeks of gestation), and four babies had a weight of less than 2500 g at birth (11.1%).

**Table 3:** Pregnancy and baby outcomes * Including two sets of twins, data for 42 pregnancy outcomes, ** Total number of mothers with live births is 36

Variables	Number (%)
Pregnancy outcome*	
Miscarriage	6 (14.3)
Live birth	36 (85.7)
Gestational age at delivery**	
< 37	4 (11.1)
37 and more	32 (88.9)
Mode of delivery **	
Cesarean delivery	13 (36.1)
Normal delivery	23 (63.9)
Baby Gender**	
Boy	20 (55.6)
Girl	16 (44.4)
Baby weight**	
< 2500 g	4 (11.1)
2500 g and more	32 (88.9)
Baby outcome**	
Normal	31 (86.1)
Congenital malformation	5 (13.9)
Minor	5(100)
Major	0

Among the 36 live babies who were exposed to betahistine in utero during pregnancy, 31 babies had normal outcomes (86.1%), five babies had minor malformations (13.9%), and none had major malformations. These minor malformations were mild pyloric stenosis, bilateral positional talipes, displaced ear, umbilical hernia, hydrocele, tongue tie, and bilateral talipes calcaneus. Exposure to betahistine was in the first trimester for the five cases. Further description for cases with malformation is shown in Table 4.

**Table 4:** Description of cases with malformation NICU: neonatal intensive care unit, IUGR: intrauterine growth restriction, CNS: central nervous system

Cases	Malformation description	Characteristics of the mother	Characteristics of the baby	Exposure to betahistine	Other exposures (covid, imaging, medications)	
Case 1	At 4 weeks of age: abdominal us showed mild pyloric stenosis. Follow up was done outside hospital (no further record)	40 years, G8P7, twin pregnancy, asthma and chronic vertigo, anemia in pregnancy	Male baby (twin B, other baby is healthy) delivered at 37 weeks via cesarean section, weight 2065 g, admitted to NICU for 3 days for low birth weight and hypoglycemia	48 mg/day, 2 prescriptions at 2- & 5-weeks’ gestation, total 40 days	COVID vaccine at 25 weeks gestation. Medications: folic acid, pantoprazole, esomeprazole, ferrous fumarate, calcium lactate, aluminum hydroxide/ magnesium hydroxide, psyllium, lactulose, metoclopramide, dydrogesterone, econazole suppository, betamethasone (2 antenatal steroids injections), xylometazoline nasal, paracetamol, mometasone nasal spray, levocetirizine.	
						Case 2	At birth: bilateral positional talipes (corrected with physiotherapy at 3 weeks)	22 years, G1P0, no co-morbidities	Female baby delivered at 38 weeks via vaginal birth, weight 2740 g	48 mg/day, 2 weeks gestation, 14 days	COVID infection at 4 weeks gestation. Medications: folic acid, thiamine, pyridoxine, ondansetrone, metoclopramide, pantoprazole, esomeprazole, nitrofurantoin, sodium bicarbonate/ tartaric acid/ citric acid/ sodium citrate, paracetamol, vitamin d, emollient		
												Case 3	At birth: low set posterior displaced ear. Follow-up at 7 weeks: small umbilical hernia, moderate hydrocele in left side, and mild hydrocele in right side, hematoma in left ear lobe	34 years, G6P4, twin pregnancy (Monochorionic - diamniotic), anemia in pregnancy (Low ferritin & low B12), mild pre-eclampsia (not on treatment)	Male baby (twin A, other baby is healthy), delivered at 36 weeks via cesarean section, weight 1660 g, admitted to NICU for 5 days for IUGR, low birth weight, hypoglycemia, subtle dismorphism	48 mg/day, 2 weeks gestation, 7 days	COVID vaccine 2 doses at 1- and 4-weeks gestation. Medications: folic acid, prochlorperazine, metoclopramide, pantoprazole, esomeprazole, paracetamol, paracetamol/orphenadrine, ferric carboxymaltose, ferrous sulfate/folic acid, cyanocobalamin, B complex, celecoxib, calcium lactate, vitamin d, levocetirizine, simethicone		
Case 4	Follow-up at 10 days: tongue tie (no further information)	29 years, G1P0, two pontine strokes (7 and 6 months before pregnancy), known case of CNS vasculitis	Male baby delivered at 38 weeks via vaginal birth, weight 3470 g	48 mg/day, 3 weeks gestation, 18 days	COVID vaccine 2 doses at 1- and 4-weeks gestation. Medications: folic acid, dabigatran, prednisolone, azathioprine, aspirin, pantoprazole, anti-d immunoglobulin, paracetamol, vitamin d, ferrous fumarate, calcium lactate/carbonate. Non-enhanced CT scan brain												
						Case 5	At birth: bilateral talipes calcaneus (corrected with physiotherapy at 4 weeks)	29 years, G3P1, no co-morbidities	Female baby delivered at 39 weeks via vaginal birth, weight 2525 g	48 mg/day, 3 weeks gestation, 21 days	COVID infection at 33 weeks gestation. Medications: folic acid, metochlorpramide, prochlorperazine, ondansetron, diphenhydramine, paracetamol, bromhexine, vitamin d, vitamin c, ferrous fumarate/sulfate/folic, multivitamin, vitamin b complex, esomeprazole, hyoscine, levocetirizine, calcium carbonate, dydrogesterone, progesterone. Non-contrast CT scan brain. MRI brain		

## Discussion

The current study provides an important insight into maternal exposure to betahistine and its possible impact on pregnancy outcomes. Despite that, betahistine is being widely used for treating vestibular disorders and vertigo; its safety profile during pregnancy remains poorly investigated. Unlike other common medications prescribed in pregnancy, betahistine has received limited attention in clinical and epidemiological research regarding maternal and fetal health outcomes. This study aimed to add to the literature to help guide clinical decision-making when prescribing betahistine to pregnant women.

In our study, no major malformations were observed in the 36 live infants that were examined. This finding aligns with a previous study that included 20 live births, which reported only one major malformation [8]. The study concluded that their results do not suggest an increased rate of major malformations associated with betahistine exposure in pregnancy [8].

Our study reported on five babies with minor malformations, which were not consistent in type between the babies. Furthermore, exposure to betahistine in the five babies was very early in pregnancy, and it was unclear whether the mother discontinued or continued taking the medication after discovering she was pregnant. The minor malformations were mild pyloric stenosis, bilateral positional talipes, displaced ear, umbilical hernia, hydrocele, tongue tie, and bilateral talipes calcaneus. All of the mothers were exposed to medications during pregnancy other than betahistine, and all of them had either received the Coronavirus disease 2019 (COVID-19) vaccine or experienced a COVID-19 infection during pregnancy, at a time when the virus was still novel to the world. Furthermore, two of the mothers were carrying twins, three mothers had co-morbidities, and two mothers needed CT scans without contrast, and one of them required an MRI during pregnancy. Two babies had a birth weight of less than 2500 g, and one baby was delivered preterm at 36 weeks of gestation. All these factors make it difficult to confirm the association between betahistine exposure and infant outcomes. Moreover, these minor malformations are not consistent with the previously published study, where patent foramen ovale was the main minor malformation reported in two infants, although the authors concluded that the data from their study does not currently support an association [8].

The global safety database for betahistine use has reported three congenital abnormalities. The first report described a girl born with partial bilateral syndactyly of the second and third toes. Her mother had begun treatment with betahistine during the third month of pregnancy, and the mother's alcohol consumption was also recorded. Another case involved a congenital abnormality suspected to be linked to mitochondriopathy. In the third report, the patient presented with radial limb defects and scoliosis after intrauterine exposure to betahistine. The complex syndrome was diagnosed as vertebral anomalies, anal atresia, cardiac defects, tracheo-esophageal fistula, renal anomalies, and limb anomalies (VACTERL) association. These reports were described as insufficient to assess the possible harmful effect of betahistine exposure during pregnancy [11].

The current study reported outcomes for a total of 42 babies, with 36 live babies who were specifically evaluated for congenital malformations. Given the limited information currently available in the literature, the findings of this research provide important insights into pregnancy outcomes and potential risks, helping to inform clinical guidance and future investigations in this underexplored area.

The study on betahistine use during pregnancy highlights several limitations that necessitate caution in interpreting its findings. First, the small number of exposed pregnancies limits the statistical power of the results. Furthermore, the small sample size along with the variability in the exposed dose and duration make it difficult to conduct dose-response analysis to assess if higher doses or longer exposure to betahistine can lead to potential teratogenic risk. Second, the retrospective nature of the reporting may introduce biases. Third, there was no assessment of maternal adherence to betahistine intake, which could affect outcomes. Fourth, incomplete information regarding other exposures during pregnancy complicates the analysis. Lastly, the study did not evaluate outcomes related to abortion cases, which could provide additional context on safety. These limitations underscore the need for well-designed epidemiological studies with larger sample sizes and robust methodologies to better assess the safety of betahistine during pregnancy.

## Conclusions

Although the current study did not identify major malformations associated with in utero exposure to betahistine, the safety of its use should be interpreted with considerable caution. Well-designed epidemiological studies involving larger sample sizes and strong methodological approaches are needed to more effectively determine the safety of betahistine during pregnancy.
